# Microsphere-assisted generation of localized optical emitters in 2D hexagonal boron nitride

**DOI:** 10.1515/nanoph-2024-0625

**Published:** 2025-06-05

**Authors:** Xiliang Yang, Dong Hoon Shin, Kenji Watanabe, Takashi Taniguchi, Peter G. Steeneken, Sabina Caneva

**Affiliations:** Department of Precision and Microsystems Engineering, 2860 Delft University of Technology, Mekelweg 2, 2628 CD, Delft, The Netherlands; Department of Electronics and Information Engineering, Korea University, Sejong 30019, Republic of Korea; National Institute for Materials Science, 1-1 Namiki, Tsukuba, Ibaraki 305-0044, Japan

**Keywords:** hexagonal boron nitride, optical emitter, microsphere, laser fabrication

## Abstract

Crystal defects in hexagonal boron nitride (hBN) are emerging as versatile nanoscale optical probes with a wide application profile, spanning the fields of nanophotonics, biosensing, bioimaging, and quantum information processing. However, generating these crystal defects as reliable optical emitters remains challenging due to the need for deterministic defect placement and precise control of the emission area. Here, we demonstrate an approach that integrates microspheres with hBN crystal lattices to enhance both hBN defect generation and optical signal readout. This technique harnesses microspheres to amplify light–matter interactions at the nanoscale through two mechanisms: focused femtosecond (fs) laser irradiation into a photonic nanojet (PNJ) for highly localized defect generation and enhanced light collection via the whispering gallery mode (WGM) effect. Our microsphere-assisted defect generation method reduces the emission area by a factor of 5 and increases the fluorescence collection efficiency by approximately 10 times compared to microsphere-free samples. These advancements in defect generation precision and signal collection efficiency open new possibilities for optical emitter manipulation in hBN, with potential applications in quantum technologies and nanoscale sensing.

## 1 Introduction

Nanoscale optical emitters are a cornerstone of nanophotonics, bioimaging, and biosensing and an invaluable tool for investigating dynamics, mechanics, and interactions of biological components in aqueous environments [1]. While traditionally relying on fluorescent dyes for biophysics research, novel probes are emerging that offer better combinations of chemical and mechanical stability, brightness, and lifetime [2]. Among next-generation probes, 2D material optical emitters (OE) based on hexagonal boron nitride (hBN) crystal defects are particularly attractive [3]. These nanoscale probes exhibit exceptional brightness (4,000 kcts/s) [4], long lifetimes (∼3 ns) [5], high quantum efficiency (87 %) [6], emission in the visible [7], stability in liquid environments [8], and biocompatibility [9], making them ideal for a wide range of applications. Unlike other 2D material-based emitters, such as transition metal dichalcogenides [10], hBN defects are fully operational at room temperature [11], [12], significantly broadening their potential in both fundamental research and applications. However, the use of hBN emitters also face several challenges: (1) Fabrication can be complex, costly, or low throughput requiring advanced techniques like ion/electron beam irradiation [13], [14], fs laser writing with high numerical aperture objective lenses, or indentation with sharp atomic force microscopy (AFM) tips [15]. (2) Controlling the nature and location of defects within the crystal structure is difficult without modifying the substrate through the addition of, e.g., nanopillars, wrinkles, or microsphere arrays, impacting reproducibility and performance [16], [17]. Addressing these challenges is crucial to integrate hBN emitters into high-resolution imaging and nanophotonics applications, necessitating further advances in fabrication and emission collection methods.

To generate OE in hBN, various fabrication methods have been developed and are broadly categorized into two groups: methods with random spatial distributions and site-specific techniques [17], [18]. The former methods include thermal annealing [19], [20], chemical [21] and plasma etching [22], and bottom-up growth [23]. These techniques typically create defects over larger areas with little or no spatial control or require nonplanar surfaces features. In contrast, spatially controlled techniques, such as AFM indentation, substrate strain-induced fabrication, ion/electron beam irradiation, and direct laser writing, offer more precise control over emitter placement [24], [25]. However, they face restrictions in terms of scalability, high-end, expensive instrumentation or require non-planar substrate engineering [24], [26], [27], [28]. Irradiation with ions also presents the risk of introducing foreign atomic species/implantation of ions [29], [30], [31]. Direct laser writing, especially using femto- to pico-second lasers, achieves precision and scalability [32], [33], yet high laser powers can lead to considerable surface roughening, caused by thermal damage over the irradiated area [34]. Additionally, the spot size cannot be reduced below the diffraction limit determined by the numerical aperture (NA) of the objective lens, which restricts the spatial precision of the fabrication process.

Here, we report an approach that utilizes a microsphere membrane to reduce the size of the irradiated area and concurrently control the position of optically generated hBN emitters. Leveraging the PNJ focusing capability of the microsphere, previously demonstrated for SiO_2_/Si and gold surfaces [35], this method achieves a fivefold reduction in the size of the area in which emitters are generated compared to fs-laser writing without microspheres, resulting in more localized hBN emitters. This improvement arises from the effective NA enhancement by the microsphere, which results in a focal spot size of <1 µm. In addition, due to the principle of reversible optical paths, microsphere-assisted fabrication significantly boosts fluorescence collection efficiency by approximately 10 times. The enhancement of the optical absorption by the defects and reduced background noise [36], [37] is further accompanied by the enhanced capture of refracted light through WGM and PNJ. In this process, light traveling around the microsphere’s surface is confined by total internal reflection, creating resonant modes that amplify and direct the light. This effect optimizes the extraction of photons into the far field, improving the efficiency of light collection and emission from the optically active hBN defect regions [38]. Practically, the microsphere traps the emitted light and directs it efficiently into a preferred out-coupling direction, further amplifying the fluorescence signal strength.

Therefore, microsphere integration not only enhances the spatial accuracy of emitter fabrication but also maximizes fluorescence signal collection. This platform can be readily integrated into single-molecule fluorescence microscopy systems, where hBN surfaces are finding increasing use as biocompatible substrates for wide-field imaging of biomolecules and their dynamics [39]. Additionally, by leveraging existing microsphere array technology [40], and the simple integration in microfluidic devices, this microsphere implementation has the potential to achieve highly parallel signal collection in optofluidic sensing for health and environmental monitoring.

## 2 Results and discussion

### 2.1 Microsphere-assisted laser fabrication

**Figure 1:**
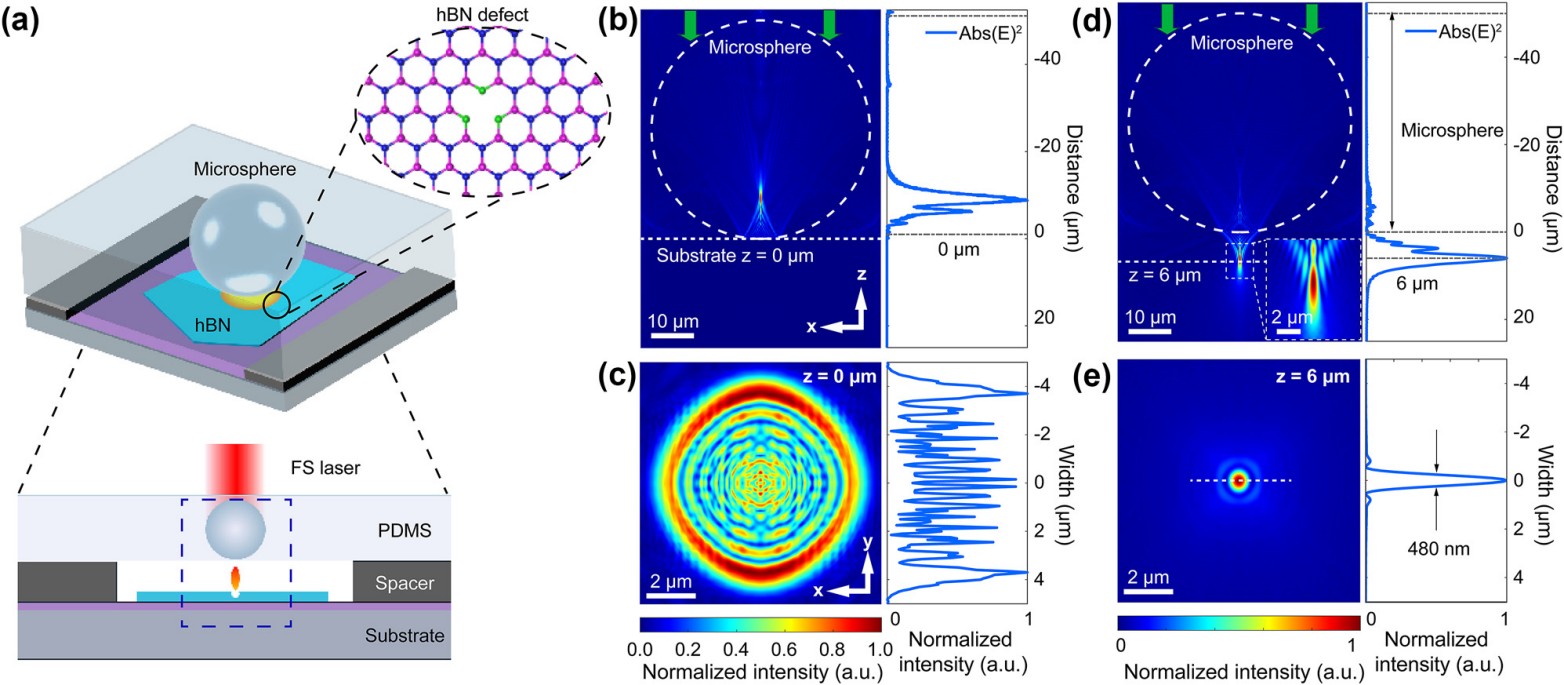
Schematic diagram of microsphere-assisted fs-laser fabrication of hBN emitters and simulations. (a) Experimental design of the MPM with spacer over the hBN flake surface on the SiO_2_/Si substrate. Bottom: Side view of microsphere enhancement of the fs-laser focus. (b)–(e) FDTD simulation of the light field distribution of the MPM focus: (b) light distribution in the *xz*-plane and intensity distribution along the *z*-axis with direct contact between the MPM and the substrate. (c) Light distribution in the *xy*-plane and intensity distribution along the *y*-axis at the maximum intensity position with direct contact. (d) Light distribution in the *xz*-plane and intensity distribution along the *z*-axis with a 6 µm distance between hBN and microsphere. (e) Light distribution in the *xy*-plane and intensity distribution along the *y*-axis at the maximum intensity position with a 6 µm distance.

Figure 1(a) shows the laser incident on the microsphere, forming a PNJ upon emerging at the bottom. The fs laser beam (515 nm, pulse duration 290 fs and linear polarization) was focused through a Theta lens. To precisely control microsphere positioning, we utilize high-refractive-index (*n* = 1.9) barium titanate (BaTiO_3_) glass microspheres (50 µm diameter) embedded in a polydimethylsiloxane (PDMS) membrane (*n* = 1.41). This design ensures three key objectives: (1) Noncontact optical focusing: leveraging the microspheres’ high refractive index and spherical geometry, the focal plane is engineered to reside externally to the microsphere, as confirmed by finite-difference time-domain (FDTD) computational modeling as shown in Figure S1 of the Supporting Information (SI). This ensures intense near-field confinement (*λ*/2.5 resolution) on hBN samples while circumventing interfacial Fresnel reflections and multiple scattering artifacts inherent to direct sphere-sample contact. (2) Deterministic alignment: the 50 µm microsphere diameter – selected to exceed twice the positional uncertainty (∼20 µm) of the fs laser translation stage – enables submicron registration accuracy during laser-induced photonic array fabrication [41], [42]. (3) Index-engineered material compatibility: the PDMS substrate simultaneously immobilizes microspheres via viscoelastic adhesion and minimizes parasitic scattering through a refractive index contrast (Δ*n* = 0.49). This contrast suppresses Rayleigh backscattering at the PDMS-BaTiO_3_ interface [43]. The composite system provides reconfigurable optical functionality while maintaining thermo-mechanical stability (PDMS glass transition temperature ≈−125 °C), ensuring reliable performance during fabrication and imaging processes. The microsphere-PDMS membrane (MPM) fabrication process is detailed in Section S2 of the Supporting Information. We note that while we focus here on single microsphere-based hBN emitter fabrication, self-assembly of microsphere during membrane embedding can generate tightly packed arrays that can increase the fabrication throughput. The MPM was placed on a SiO_2_/Si substrate on which mechanically exfoliated, high-quality hBN flakes were transferred. To ensure the focus point could reach the hBN surface, we inserted a spacer consisting of double-sided tape between the MPM and the substrate. To determine the changes in optical field distribution caused by the microsphere, finite-difference time-domain (FDTD) simulation designs were conducted. The results show that when the MPM directly touched the substrate, it produced ring-shaped patterns due to focusing inside the microsphere, as shown in Figure 1(b) and (c). Increasing the distance between the hBN and the microsphere improved PNJ focusing, as shown in Figure 1(d) and (e), with a full-width at half-maximum (FWHM) of 480 nm at a distance of ∼6 µm from the microsphere, which is smaller than the focal spot size of most high-NA objective lenses. Additionally, the PNJ length was approximately 5 µm, providing a large working distance tolerance for fabrication.

**Figure 2:**
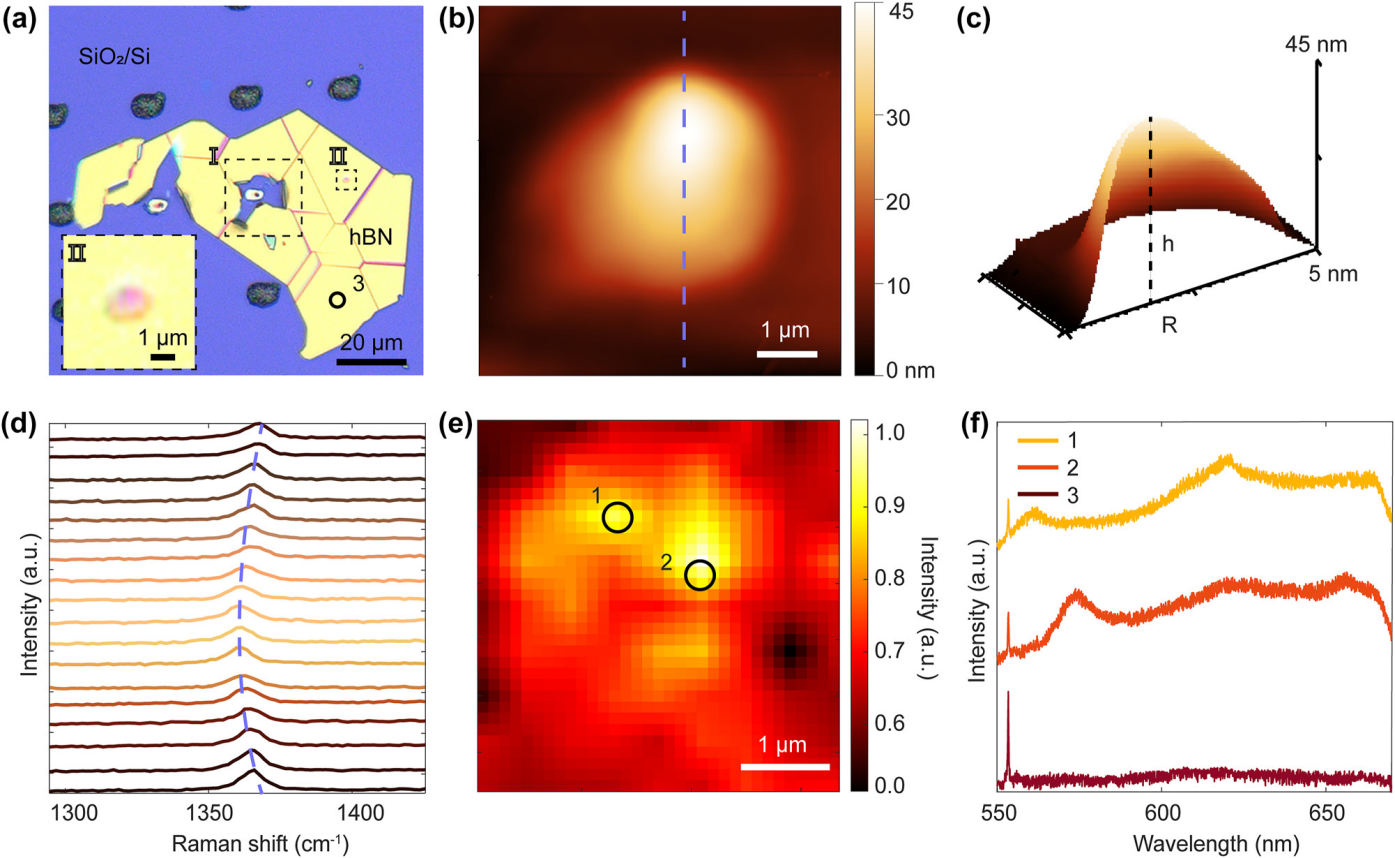
Characterization of fs-laser induced hBN defects. (a) Optical image of the hBN surface after fs-laser irradiation with (area II) and without (area I) MPM (inset: zoomed view of the MPM irradiated area, scale bar: 1 µm). (b) 2D AFM image of the hBN bubble. (c) 3D AFM image of the bubble cross section. (d) Raman spectra along the diameter of the bubble (highlighted in (b) by a purple dashed line). (e) PL mapping of the hBN bubble. (f) Emission spectra of two representative emitters and background, labeled as circles 1 and 2 in (e), and 3 in (a), respectively.

Based on the stack design and simulations in Figure 1, fs-laser fabrication was used to generate defects in hBN using a 6-µm spacer, the profile of which is depicted in Figure S3. The irradiation spots on the *xy*-plane were spaced approximately 30 µm apart to avoid crosstalk. Figure 2(a) compares the results of fs laser processing without and with microspheres. Area I shows that during direct fs-laser writing, the hBN layer (yellow) is fractured, producing an irregular opening of ~15 μm that exposes the SiO_2_/Si substrate (blue) underneath it. When the laser pulse is applied directly to the SiO_2_/Si, holes are formed in the surface (black). . Strikingly, on the same flake, laser writing with the MPM layer reduces the affected area (area II) to a diameter of approximately 3.6 µm. This reduction in diameter corresponds to a decrease in the affected area by a factor of ∼5. The hBN forms a bubble that exhibits a circular shape, with a radius of 1.8 µm and a height of 45 nm (*h*/*R* = 0.025), as shown by the AFM analysis (Figure 2(b) and (c)). This observation suggests that a tightly focused PNJ causes deformation of the top layers of the hBN flake (*t*_hbn_ ∼ 50 nm), caused by localized heating from high-intensity laser pulses, which leads to thermal expansion and blistering [35]. Bubble formation in 2D materials has been previously attributed to mechanical stress from rapid heating and cooling, which can trap air as well as ambient or surface absorbents [44], [45]. The bubble’s ratio *h*/*R* = 0.025 here is lower than that of monolayer hBN bubble formation (*h*/*R* = 0.11) [46] but is in good agreement with the multilayer hBN bubble formation seen in both experimental and theoretical results [45].

**Figure 3:**
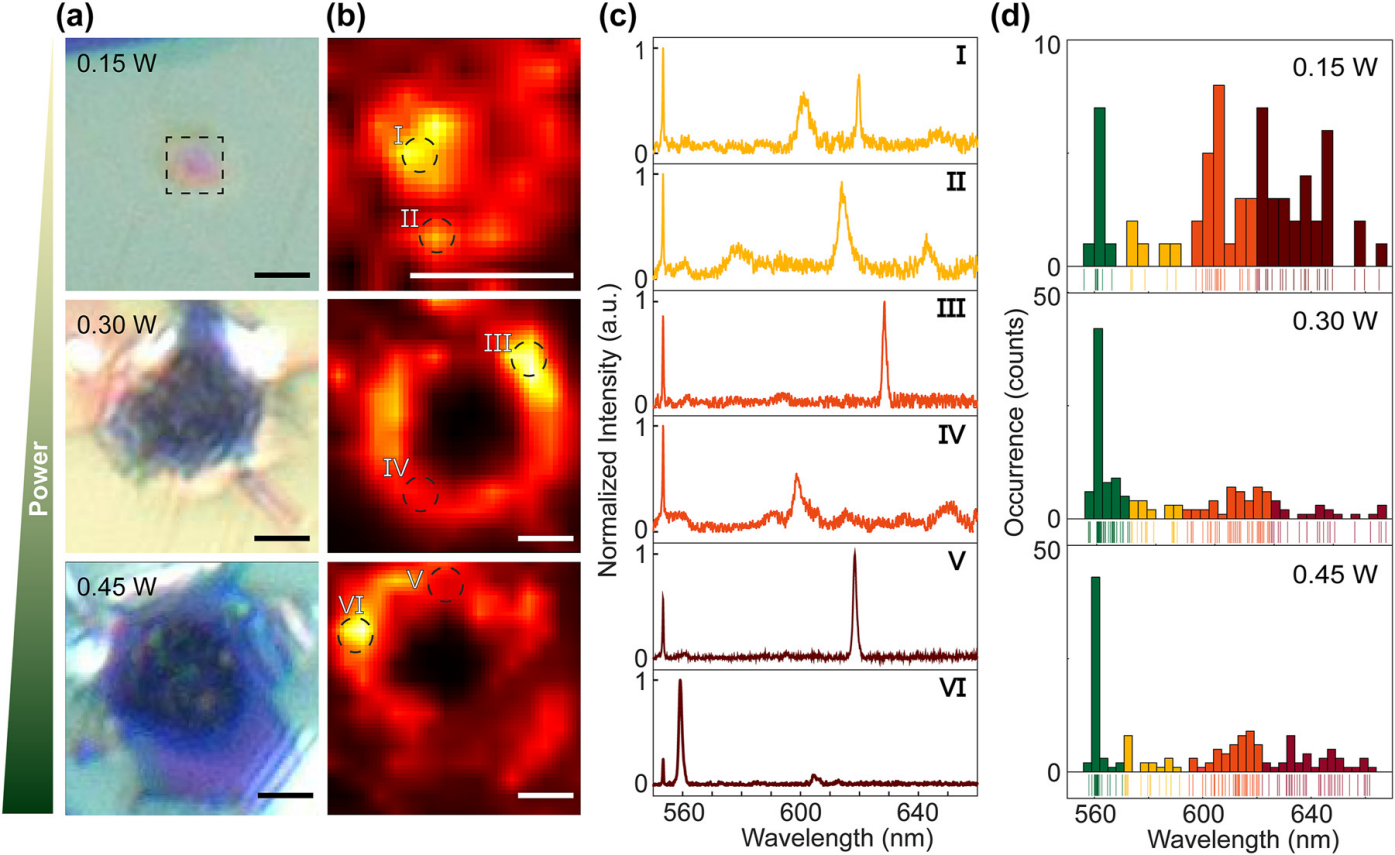
Optical and spectral characterization of MPM fs-laser-generated emitters in hBN. (a) Optical images of the defects of different sizes generated by MPM fs-laser with powers of 0.15, 0.3, 0.45 W. Scale bar: 3 µm. (b) The corresponding PL maps of the irradiated areas in (a). Scale bar: 3 µm. (c) Emission spectra of six representative emitters, as labeled in (b). The 550 nm peak in all panels arises from the hBN Raman contribution. (d) Histograms of the emitters ZPLs, categorized by different color ranges. Bin size: 3 nm.

Figure 2(e) shows the photoluminescence (PL) of the hBN bubble, with most emitters located at the edges of bubble, where the strain is most pronounced. This suggests that the emission is largely dependent on lattice strain, as evidenced by a Raman hBN peak shift from 1,365 to 1,363 cm^−1^ across the bubble as shown in Figure 2(d), indicating tension in the hBN flake [45]. Similar peak shifts due to maximized strain at the bubble’s center have previously been reported [45]. Figure 3(f) displays the PL spectra from these bright spots (circles 1 and 2 in Figure 2(e)), showing the typical zero-phonon lines (ZPL) of hBN emitters.

Figure 3 demonstrates different sizes of defects produced with MPM by varying the laser irradiation power. When the power was below the surface modification threshold (∼0.07 W) in our setup, slight color changes are visible in the hBN, but no clear defect areas could be observed. However, at higher power levels, clear visible structural changes appeared in the material. Details of the laser-induced defects are shown in Figures 3(a) and S4.

When the laser power exceeded the surface modification threshold but remained below 0.3 W, bubble patterns were observed. The diameter of these bubble structures ranged from 3 to 4 µm, which was smaller than the focal spot size of the f-theta lens (9 μm at 1/e^2^ intensity), as shown in Figures 2(a), 3(a), and S4. This size reduction can be attributed to the PNJ generated by the microsphere, which effectively reduces the focal spot size. Pulses with power above 0.3 W caused ablation at the center of the PNJ, resulting in the outward redeposition of hBN material around the hole. With 0.3 W and 0.45 W laser power, the defect patterns generated by MPM enlarged to 4.5 µm and 5.6 µm, respectively, breaking up into holes. In comparison, direct laser fabrication patterns were about 5 times larger than those produced by the MPM method as shown in Figure S4(e). The defect patterns exhibited torn edges, likely formed by the high pressure and shock waves generated by the high-energy laser pulses, accompanied by a breakdown of the surrounding material, similar to laser-induced micro explosions confined within the bulk of sapphire [35], [47]. Additionally, we confirm that the SiO_2_/Si substrate exhibits no intrinsic fluorescence, ensuring that hBN defect emissions are free from background interference as shown in Figure S5. The average area of the defect patterns, based on over 15 samples, increased linearly with the pulse power with a scaling factor of 13 μm/W, as shown in Figure S4(e).

We note that the observed variation in the size of the defect patterns, even with the same pulse energies, can be attributed to factors such as the variation in hBN flake thickness (*t*_hBN_: 40 nm–70 nm), the concentration of impurities or defects in the bulk material of hBN, wrinkles formed during exfoliation, and misalignment of the laser writing setup and the microsphere as shown in Figure S6 [15], [48]. These factors also prevent the formation of smaller defects as predicted by simulations.

To activate and stabilize the photoemission from the hBN defects and remove the contamination induced during the fabrication process, the hBN samples were annealed in 1,000 °C at 10^−7^ bar for 2 h after laser writing with the MPM. As previously reported, annealing plays an important role in restructuring and forming new optically active color centers in hBN [33]. Before annealing, few PL signals were observed from the laser-processed sample. After annealing, sharp, bright PL peaks appeared at laser-irradiated sites as shown in Figure 3(c), unlike emitters produced by conventional direct thermal annealing methods, which are located on randomly generated wrinkles [20]. PL maps were subsequently obtained on these processed areas using a 0.9 NA objective with a 514 nm wavelength continuous-wave laser excitation (Figure 3(b)).

The maps indicate that multiple emission centers are generated and distributed along the edge of the affected areas. Moreover, the number and type of emitters changed with the defect pattern size. Single and double emission spots can be seen around the defect pattern in the bubble structure, yet due to the diffraction limit of the optical system, we cannot identify and determine the number of individual nanoscale emitters. When the pattern size exceeded ∼3 µm, the emission spots started to merge into clusters, as shown in the middle panel of Figure 3(b).

With 0.15 W laser power, the formation of bubble structures introduces localized strain in the hBN’s crystal lattice, distorting molecular orbitals and perturbing the energy levels of defect states [11]. This strain-induced modulation enhances the possibility of emitters exhibiting overlapping zero-phonon line (ZPL) wavelengths, as lattice distortions can stabilize defects with similar electronic configurations [49]. While our analysis of 12 representative bubble-structured samples reveals ZPL emissions spanning all categorized ranges (green: 555 ± 15 nm, yellow: 580 ± 10 nm, orange: 605 ± 15 nm, red: 650 ± 30 nm), a statistical preference for orange (605 ± 15 nm) and red (650 ± 30 nm) ZPLs is observed as shown in Figures 3(d) and S7. This trend suggests that strain fields in bubble structures preferentially stabilize defects associated with longer wavelengths, such as N_B_V_N_ defects [5], [11], [13]. Strain magnitudes and lattice distortions vary spatially throughout the bubble geometry. This spatial heterogeneity results in a nonuniform distribution of defect patterns, where specific defect types dominate in regions that match their stable strain conditions. As a result, the zero-phonon line (ZPL) energies of these defects show a broad spectral distribution, reflecting the structural diversity of defect configurations within the bubble. This phenomenon is consistent with defect aggregation observed during low-power laser fabrication, where localized energy deposition and rapid thermal gradients promote the aggregation of vacancies, displacements, and strain-stabilized complexes.

As the laser power increases from 0.15 W to 0.45 W, the ZPL full width at half-maximum (FWHM) narrows by 1.1 nm, indicating a transition from a diverse strain-tuned defect landscape to a more uniform vacancy-dominated state. This suggests that higher laser power disrupts strain-stabilized defects, favoring the formation of vacancy-rich sites with well-defined electronic states. For defect patterns obtained with 0.45 W irradiation, the ZPLs are narrow and sharp, comparable to other emitter fabrication methods (e.g., FIB, AFM indentation) [14], [24].

At 0.45 W laser power, the accumulated thermal stress exceeds the structural tolerance of hBN, rupturing the initial bubble-like defects and creating holes with fractured edges (Figure 3(b)). This transition is accompanied by significant surface damage, dominated by atomic-scale vacancies at rupture sites. These vacancy-type defects predominantly emit in the shorter-wavelength regime (∼560 nm, Figures 3(c) and S7), consistent with reports attributing 550–590 nm ZPLs to nitrogen vacancies in hBN [5], [50]

Statistical analysis (Figure S7) reveals a pronounced spectral shift: while bubble structures (low-power regime) host emitters with ZPLs clustered at 600–640 nm, the high-power regime (0.45 W) produces a dominant 550–590 nm emission band, with fewer than 10 % of emitters exceeding 600 nm. This power-dependent transition aligns with prior studies demonstrating that subablation fs laser energies generate strain-tuned bubbles, whereas near-ablation thresholds produce vacancy-rich holes [12]. This stark contrast underscores the role of laser power in modulating defect populations: low-power regimes stabilize strain-introduced defects with longer ZPLs, while high-power ablation generates vacancy-rich sites with shorter ZPLs.

### 2.2 Microsphere-enhanced optical signal collection

**Figure 4:**
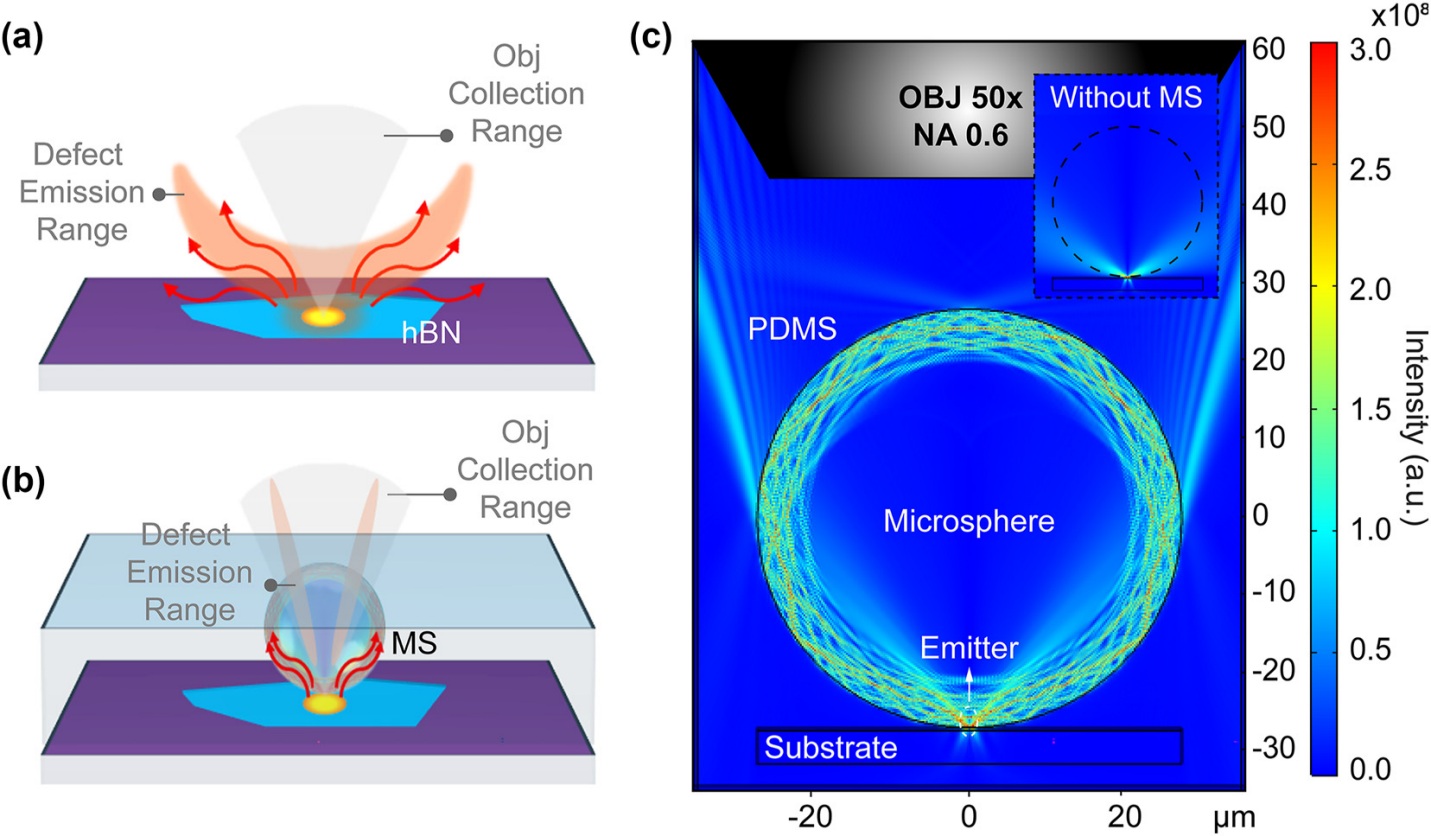
Schematic of microsphere-enhanced fluorescence collection. (a) Illustration of the spatial emission distribution of hBN emitters without microsphere. The orange area shows the defect emission range, and the red arrows indicate the extreme angle of defect emission (we note that it will be in 3D). (b) Illustration of the spatial emission distribution of hBN emitters with a microsphere, where emitted photons are coupled to the microsphere via WGM and re-radiated at an engineered angle that falls within the collection range of the objective lens (light gray area). (c) Simulation of optical WGM in the MPM system, enhancing the fluorescence signal of the emitters. The inset shows the spatial emission distribution of an emitter without a microsphere.

Beyond using microspheres to deterministically generate hBN optical emitters with a tightly focused PNJ and reduced irradiated area, we further demonstrate that the same MPM layer can be employed as a versatile component for enhancing optical signals. As shown in Figure 4, the MPM significantly boosts the intensity of both excitation and emission signals. This is not solely due to the focusing effect, but results from two key factors: the efficient coupling of both near-field and far-field energy from the hBN optical emitters, and the additional enhancement provided by WGM, which together improve the transmission to the subsequent optical components. This enhancement allows optical emitters to be imaged with low NA objectives (NA = 0.6, 50×) without requiring an oil-immersion high NA lens (NA > 1), enabling high-resolution imaging with a simpler, lower-cost optical setup.

**Figure 5:**
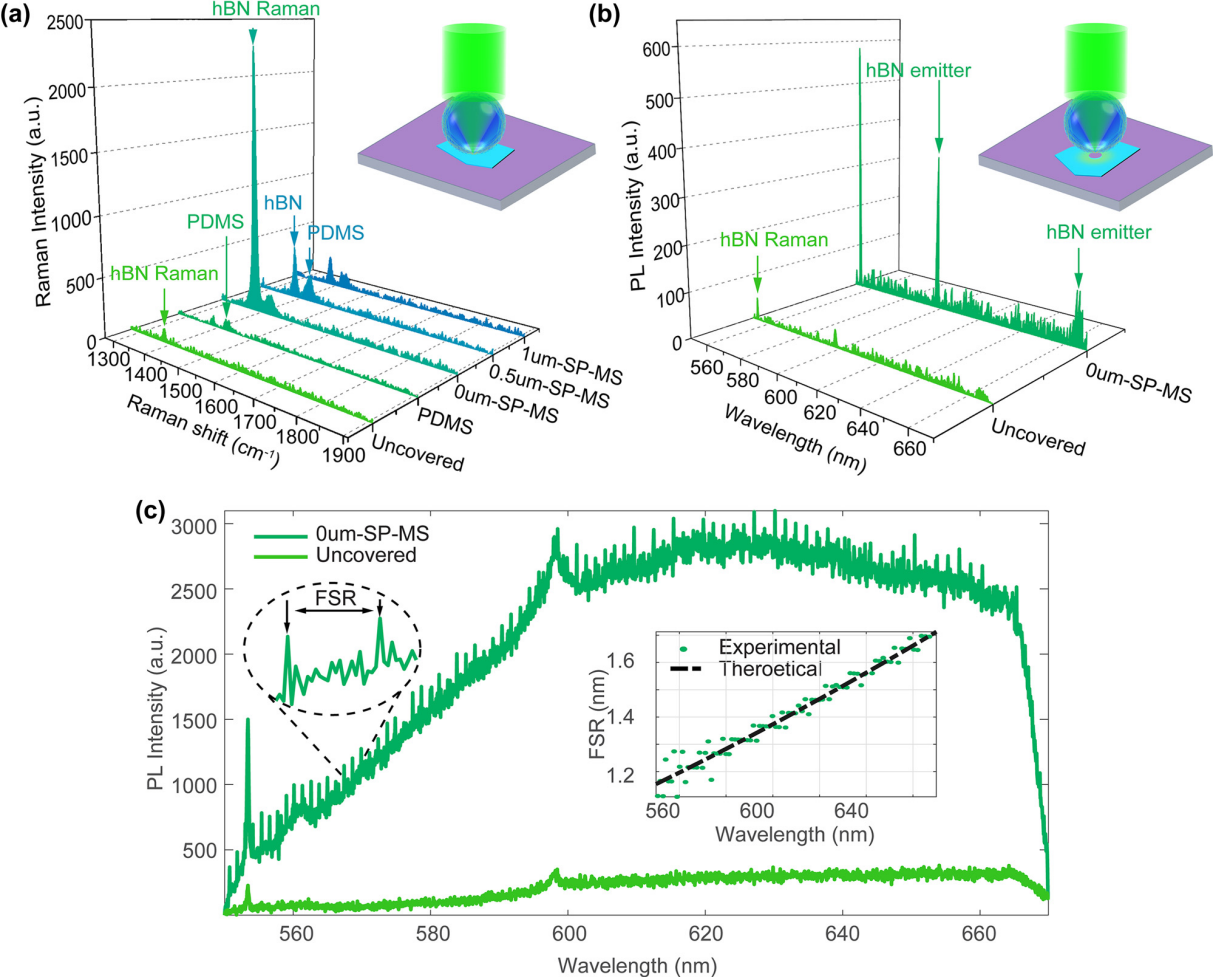
Signal enhancement from hBN flakes covered with MPM. (a) Processed Raman spectra of an hBN flake enhanced by MPM with varying spacer distances. Inset: Schematic of the Raman measurement setup for the hBN flake covered by MPM. (b) Processed PL spectra of hBN emitters fabricated via MPM fs-laser, comparing MPM-covered and uncovered cases. Inset: Schematic of the PL measurement setup for emitters at the hole edge. (c) Raw PL emission spectra of WGM-regulated hBN emitters. Left inset: Zoom in on PL spectra highlighting enhanced peaks and FSR. Right inset: Experimental versus theoretical FSR evolution across wavelengths.

The incorporation of the MPM modifies the transmission and distribution of light emitted by the optical emitters (Figure 4(a) and (b)), resulting in notable enhancements in signal intensity and imaging resolution. These improvements are driven by two key mechanisms:

(1) Spatial localization of the excitation area: The MPM confines the laser focus to the submicron scale (Figure 1(g)), concentrating energy on a smaller, well-defined three-dimensional region. This enhanced focus increases absorption efficiency at defect sites while minimizing excitation in nontarget areas, thereby reducing background noise and preventing unwanted excitation of neighboring emitters. Consequently, the overall excitation efficiency of the desired emitters is significantly improved [51], [52].
(2) Enhancement of the signal extraction efficiency: When emitters are excited outside the plane of the substrate and directly coupled into the MPM (Figure 4(c)), the light is refracted and an enhancement arises from two complementary mechanisms: (1) WGM: these trap Rayleigh-scattered light within the microsphere, resonantly amplifying emission rates via high-Q cavity effects [36]. This is evidenced by narrow spectral peaks, free spectral range (FSR) periodicity, and the refractive index (n)-dependent FSR trend (Figures 5(c), S8, and S9); (2) PNJ, which not only enhances excitation efficiency by focusing incident light but also improves collection efficiency by redirecting scattered light toward a low-NA objective. While WGMs dominate the spectral features, nanojets facilitate efficient near-field to far-field light convergence. This dual mechanism contributes to overall signal enhancement, particularly in broadband emission, thereby significantly increasing the far-field photon extraction rate [53]. COMSOL simulations indicate that this enhancement exceeds 4,000 times at the top of the microsphere, as shown in Table S1. This mechanism optimizes the propagation path of photons, improving their output efficiency and enhancing the fluorescence signal’s intensity and stability [26].

Through the synergistic effect of these mechanisms, the MPM can be used to significantly enhance the optical detection of hBN emitters.

The MPM with a single microsphere was first employed to quantitatively estimate the enhancement ratio by enhancing the hBN Raman peak. This measurement requires less localization since the Raman peak can be detected anywhere on the hBN flake surface. A laser beam was focused using a 50× objective with an NA of 0.6 onto the center of the microsphere to achieve maximum enhancement. Figure 5(a) shows the Raman spectra obtained using MPM with different gaps: 1 µm spacer with microsphere (1 µm-SP-MS), 0.5 µm spacer with microsphere (0.5 µm-SP-MS), and 0 µm spacer with microsphere (0 µm-SP-MS). Two control groups were also included: one with only PDMS and another uncovered hBN. With two control groups, we can determine the original intensity of the Raman peak on the flat hBN surface, and the peak contributed by PDMS.

For MPM without a gap, the hBN Raman peak showed approximately a factor 20 enhancement compared to the case without MPM, but the enhancement factor decreased to ∼4 and ∼2 for 0.5 µm and 1 µm gaps, respectively. This demonstrates that most of the photon energy is lost in free space without microsphere-enhanced collection within these short distances. This also clarifies why, despite simulations predicting a 4000-fold enhancement, real experiments achieve lower values, largely due to the challenge of perfectly aligning the emitter at the exact center of the microsphere’s focal region (Figure S8(b), points A and B). As a result, some enhancement is lost. Thus, achieving close attachment of the MPM to the surface is crucial for maximizing emission collection efficiency.

We note that while the same MPM chips can be used for both fabrication and collection at an intermediate distance of 0 and 6 µm between the microsphere and the substrate, the performance will be slightly compromised due to this gap size. As shown in this study, varying this gap can optimize performance depending on the desired application, providing flexibility in choosing the most suitable gap size for either extraction or collection.

Next, the MPM was attached directly to an emitter at the hBN flake edge, created via microsphere fs-laser writing. The microsphere serves as a small lens, forming a virtual image of the sample surface [54], allowing us to precisely localize the emitters at the edge (inset, Figure 5(b); optical image in Figure S10). This ensures the MPM overlaps with and enhances emission from the region of interest.

The MPM is mainly composed of BaTiO_3_, which has a wide bandgap energy (∼3.2 eV) and does not support low-energy excitons, resulting in negligible optical loss in the visible wavelength band [55]. This property makes BaTiO_3_ an excellent optical dielectric, enabling efficient light transmission and manipulation. In Figure 5(b), the PL spectra of the hBN emitter collected with (dark green) and without (light green) the MPM show a significant enhancement at 598 nm, with the MPM amplifying the PL signal by approximately a factor of 10.

As shown in Figure 5(c), the periodic narrow peaks in the raw PL spectra confirm WGM contributions to fluorescence enhancement, with spectral intervals FSR matching the theoretical model for dielectric microspheres: *FSR* ≈ *λ* ^2^/2*πRn*_eff_, where *λ* is the emission wavelength (550–750 nm), *R* is the microsphere radius, and *n*_eff_ is the effective refractive index, which is approximated as $\sqrt{\left(n^{2}+n_{\text{medium}}^{2}\right)/2}$ , where *n* and *n*_medium_ are the refractive indices of the microsphere (BaTiO_3_ glass, *n* = 1.90) and surrounding medium (PDMS, *n* = 1.41). The correlation coefficient (>0.98) between experimental and theoretical FSR across wavelengths (Figure 5(c), inset) confirms the WGM origin of the spectral periodicity.

Systematic studies (Figure S9) demonstrate direct modulation of FSR by different refractive index microspheres: for a microsphere with a given size (*R* = 25 µm), switching from SodeLime Glass microspheres (*n* = 1.5) to BaTiO_3_ glass microspheres (*n* = 1.9 and *n* = 2.2) reduces the FSR from 1.85 nm to 1.39 nm at 61 nm, consistent with the inverse dependence on *n* in the WGM mode. The raw spectral data in Figure 5(c) and refractive-index-dependent trends in Figure S9 collectively validate the role of WGMs.

However, the PL peak enhancement ratio is lower compared to the Raman peak. Since Raman intensity scales with the fourth power of the electric field (*E* ^4^), while PL scales with *E* ^2^, the Raman signal benefits more from the enhanced field, leading to a higher enhancement ratio [56], [57]. Additionally, slight spatial misalignment between the emitter and the BaTiO_3_ microsphere could create a gap as shown in Figure S5(b), allowing some surface waves, such as evanescent waves, to be lost into space rather than being collected by the microsphere and subsequent optical systems. These factors may contribute to the reduced enhancement. Despite these factors, the observed PL enhancement demonstrates the effectiveness of the MPM in improving signal strength, making full use of BaTiO_3_’s favorable optical properties.

## 3 Conclusions

In summary, our study demonstrates a new route enabling the combined fabrication of hBN emitters and fluorescence emission enhancement by using a microsphere chip as an effective and low-cost focusing lens. By combining the MPM with a fs-laser writer setup, we achieve a fivefold reduction in the irradiated area, leading to better localization and higher quality emitters (i.e., smaller FWHM of the ZPLs) in hBN, and suppressing the extensive damage found in microsphere-free fabrication. This approach not only enables better control over the hBN defect generation process but also substantially improves optical signal collection efficiency by approximately 10 times compared to microsphere-free measurements methods. The enhancement in defect absorption, combined with optimized photon extraction and efficient light directionality imposed by the microsphere geometry, results in significantly stronger optical signal detection. We emphasize that this proof-of-concept emitter fabrication and emission collection with microsphere can be highly parallelized through the use of self-assembled microsphere arrays over large-area hBN surfaces. The MPM can also be integrated in microfluidics, enhancing the detection of fluorescent biomolecules in physiological conditions. Furthermore, our findings can be readily applied to other 2D materials exhibiting optically active defects, setting the stage for further developments in nanophotonics and fluorescence imaging at 2D material surfaces.

## 4 Methods

### 4.1 Sample preparation and laser irradiation

Multilayer hBN flakes were first mechanically exfoliated from bulk hBN crystals produced by high temperature and high-pressure synthesis (NIMS Japan) and then transferred onto SiO_2_/Si surface cleaned by ultrasonic sonication in acetone and IPA for 3 min and oxygen plasma cleaning for 5 min. Using atomic force microscopy (AFM, Cypher), large-area hBN thin flakes with thickness of ∼50 nm were selected. We use high-refractive index (*n* = 1.9) barium titanate microspheres (diameter = 50 µm, Cospheric, USA) embedded in PDMS (SYLGARD 184 Silicone Elastomer Kit, Germany) to generate the MPM. We inserted a ∼6 µm spacer consisting of double-sided tape (Zhuanyi Electronic Elechnology Co., SuZhou, China) between the MPM and the substrate.

Subsequently, a single fs-laser pulse (Pharos PH1-15, Light Conversion) of wavelength 515 wavelength, pulse duration 290 fs and linearly polarization was focused through a f-theta lens (*F* = 100 mm, 510–550 nm Fused Silica Telecentric, VONJAN). The objective lens was mounted on a piezo nano positioning stage (PI E665) with nanometer resolution to precisely control the focal position. After laser irradiation, the hBN samples were annealed in a tube furnace at 1,000 °C in vacuum (4.6E-07 mbar) for 2 h.

### 4.2 Optical measurement

Raman and PL spectra were acquired in Renishaw Raman setup. The hBN samples were excited by a 514 nm argon laser (MODU-LASER) with 0.15 mW. The spectra were collected with an objective lense (Olympus 50×) with numerical aperture (NA) of 0.6 and a 1800 l/mm grating. PL mapping was performed with the same setup and excitation laser, but using a higher-resolution objective (Olympus 100×, NA 0.9) and a scanning step of 0.5 μm.

### 4.3 Photonics simulation

Numerical simulations of the electromagnetic field were conducted using a finite-difference time-domain (FDTD) method in Ansys Lumerical FDTD for focusing analysis, and a finite element method (FEM) in COMSOL Multiphysics for optical WGMs. The simulations were performed over an area of 60 µm × 30 µm with a mesh size of *λ*/100. Perfectly matched layers and periodic boundary conditions were applied to ensure accuracy, and the boundary matching layers were set as perfect absorption layers. The refractive indices of the PDMS film and microspheres (diameter = 50 µm) were set to 1.41 and 1.9, respectively, with the incident light as a plane wave at 514 nm wavelength. The simulation conditions were consistent with those of the experiment.

## Supplementary Material

Supplementary Material Details
