# Supplementary material for: Microsphere-assisted generation of localized optical emitters in 2D hexagonal boron nitride
Source: Nanophotonics. 2025 Jun 5;14(14):2419–30. doi: 10.1515/nanoph-2024-0625 (PMC12273537; doi:10.1515/nanoph-2024-0625)
Supplement: Supplementary file 1 — Supplementary Material Details [file j_nanoph-2024-0625_suppl_001.docx]

Supplementary Material

**Microsphere-assisted generation of localized optical emitters in 2D hexagonal boron nitride**

Xiliang Yang,^†^ Dong Hoon Shin,^±^ Kenji Watanabe,^§^ Takashi Taniguchi,^§^ Peter G. Steeneken,^†^ Sabina Caneva,^†^*

† Department of Precision and Microsystems Engineering, Delft University of Technology, Mekelweg 2, 2628 CD, Delft, The Netherlands.

± Department of Electronics and Information Engineering, Korea University, Sejong 30019, Republic of Korea.

§ National Institute for Materials Science, 1-1 Namiki, Tsukuba, Ibaraki 305-0044 Japan

* Corresponding author. Email: [s.caneva@tudelft.nl](mailto:s.caneva@tudelft.nl)

**The supplementary information includes:**

S1: Focusing characteristics of microspheres with varying refractive indices

S2. Fabrication of the microsphere-PDMS membrane film.

S3. Height profile along the edge of the tape spacer.

S4. Power-dependent defect formation in fs laser fabrication with and without microspheres

S5. Optical and epi-fluorescence images of fs laser-processed regions on SiO₂/Si and hBN flakes.

S6. Aligned and misaligned microsphere-assisted fs laser patterning on the hBN flakes.

S7. Percentage distribution of ZPL wavelengths with increasing fabrication power.

S8. Simulated electric field distribution for the emitter collection.

S9. WGM Tuning in Microspheres with Varying Refractive Indices for PL Enhancement.

S10. Simulated electric field distribution on the microsphere.

S11. Optical images at different image planes of the microsphere.

**S1: Focusing characteristics of microspheres with varying refractive indices**


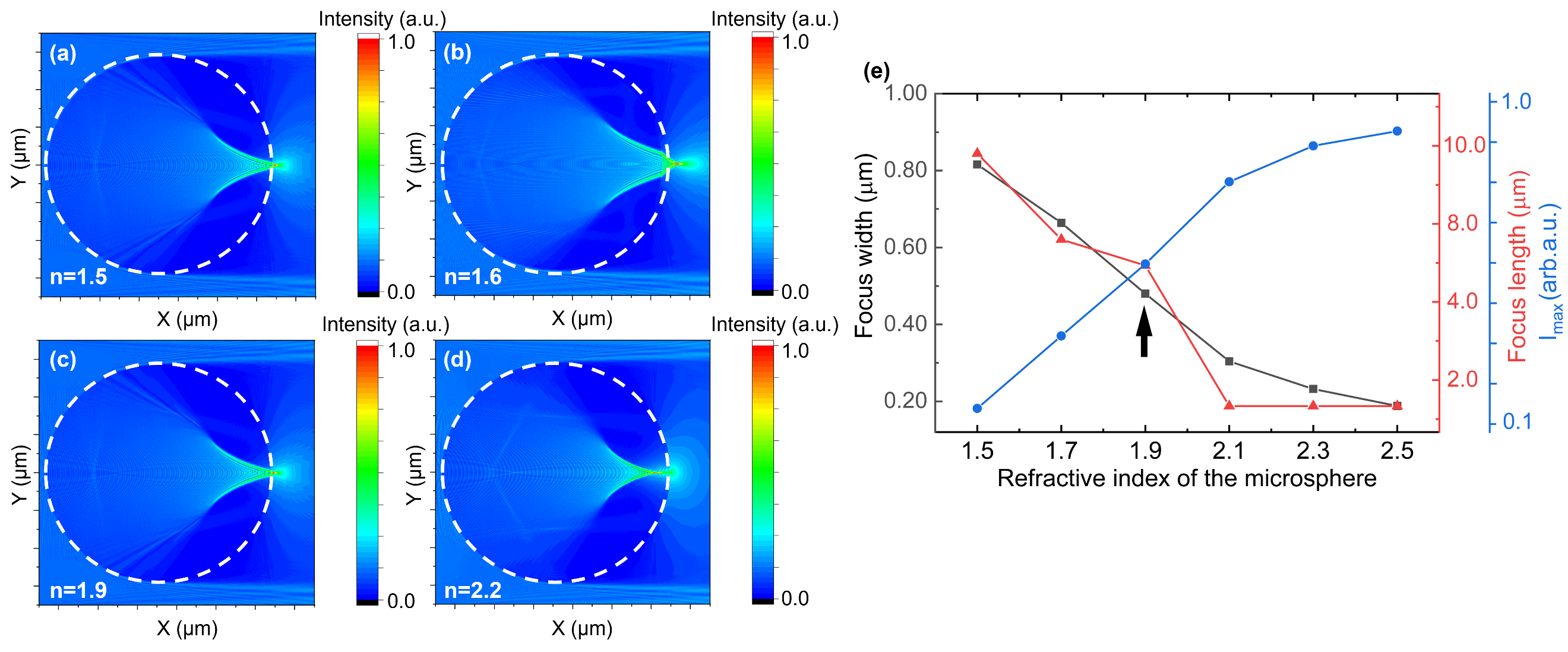


**Figure S1.** Focusing characteristics of 50 μm microspheres with varying refractive indices. (a-d) Focusing behavior for microspheres with n = 1.5, 1.6, 1.9, and 2.0. (e) Influence of refractive index on focal length, focal width, and intensity ratio (maximum/original intensity) when immersed in PDMS.

**S2: Fabrication of microsphere-PDMS membrane film**

The experimental procedure to generate the MPM is outlined in Figure S1 below.


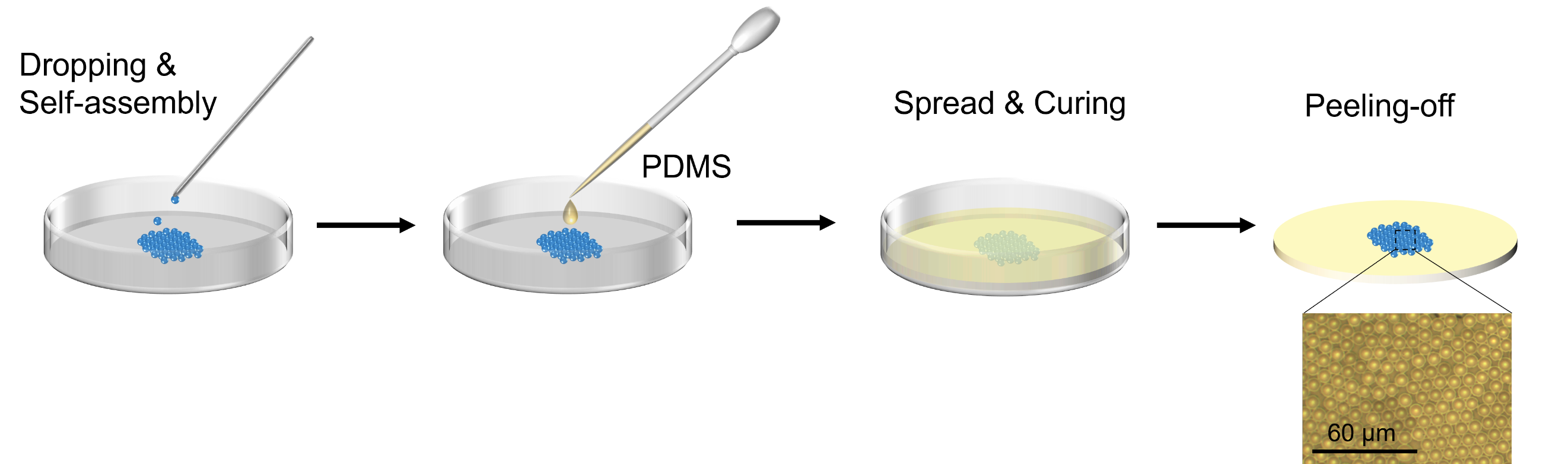


**Figure S2.** Fabrication of microsphere-PDMS membrane film by microsphere dropping and self-assembly, PDMS spreading and curing with microsphere beads, and pealing-off the microsphere film by the tweezer. Insert figure: optical image of microsphere-PDMS membrane film.

**S3. Height profile along the edge of the tape spacer**


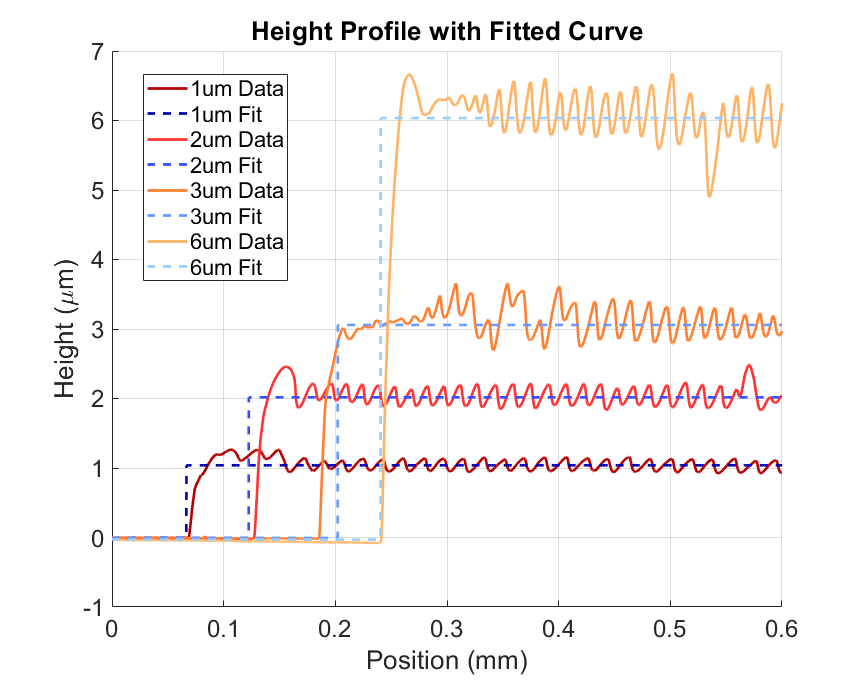


**Figure S****3.** Profilometer measurement of the spacer (double-side tape) with different thicknesses. The sticky upper surface of the double-sided tape causes some stickiness in the scanning tip, leading to a less smooth scan result. Despite these variations, the tape’s thickness we used, approximately 6 microns, is still clearly discernible.

**S4. Power-dependent defect formation in fs laser fabrication with and without microspheres**


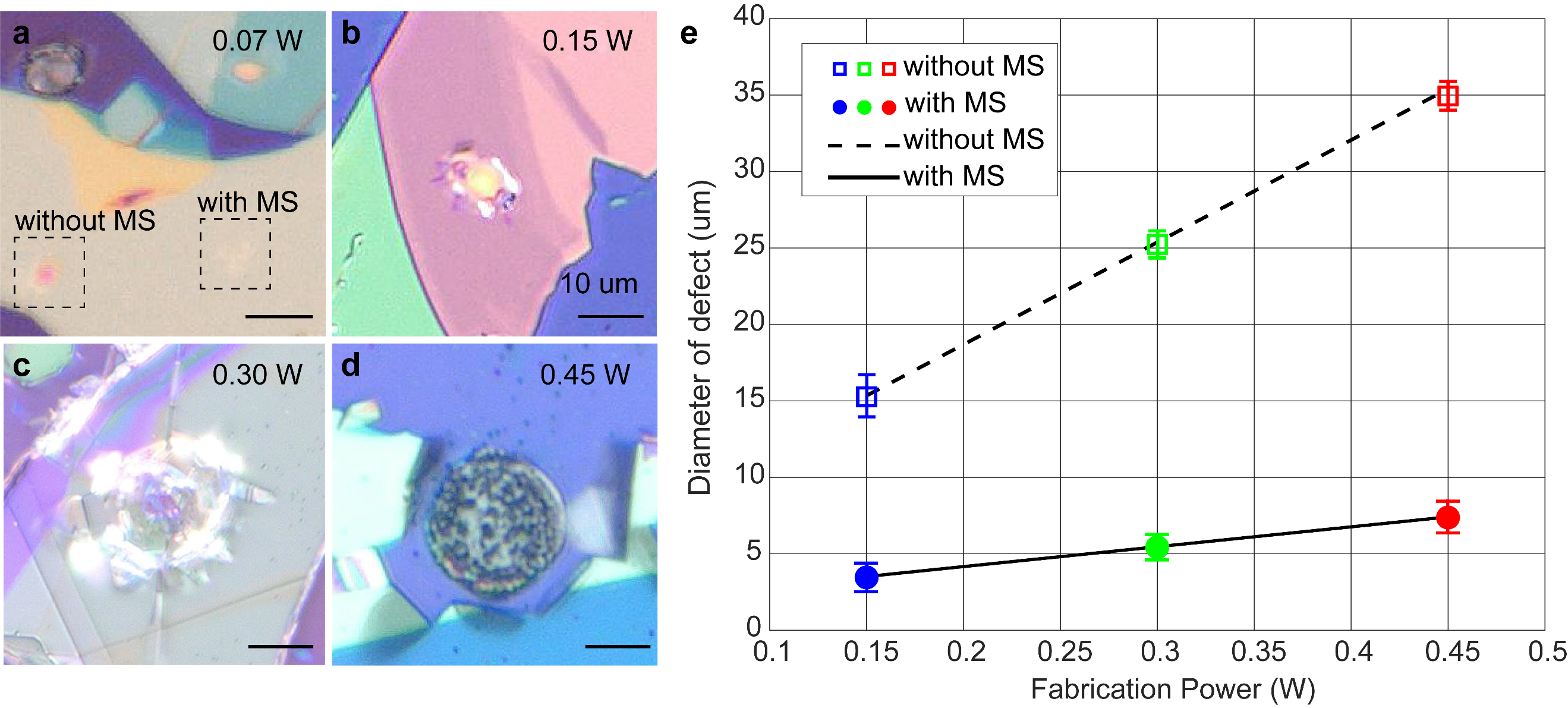


**Figure S4.** Optical images of defects created by direct fs laser fabrication with varying power levels: (a) 0.07 W, (b) 0.15 W, (c) 0.3 W, and (d) 0.45 W. Scale bar: 10 µm. (e) Diameter of defects vs fs laser power for both with and without microspheres. Data from 15 or more defects at each power level are included. The dashed line represents a linear fit to the diameter of defects without microspheres, with a 66.87 µm/W slope. The dashed-dotted line represents a linear fit to the diameter of defects with microspheres, with a slope of 13 µm/W.

**S5. Optical and epi-fluorescence images of fs laser-processed regions on SiO₂/Si and hBN flakes**


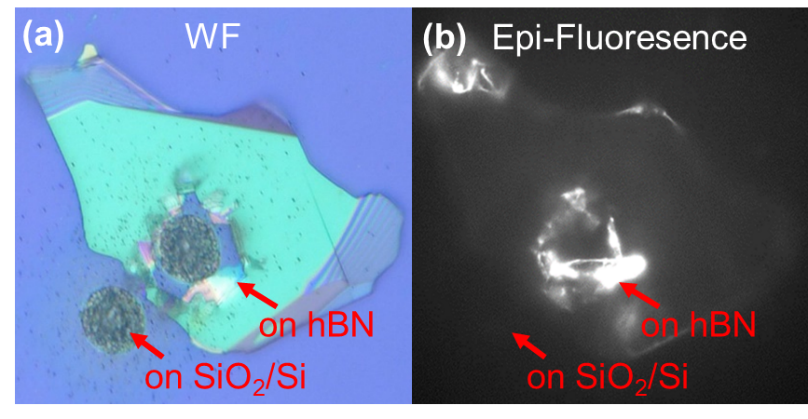


**Figure S5.** Optical and epi-fluorescence images of fs laser-processed regions on bare SiO₂/Si and hBN flakes. (a) Optical image of processed regions on bare SiO₂/Si (left) and hBN flakes (right). (b) Epi-fluorescence image showing localized emission from hBN defects with no background fluorescence from SiO₂/Si.

**S6. Aligned and misaligned microsphere-assisted femtosecond (fs) laser patterning on the hBN flakes**


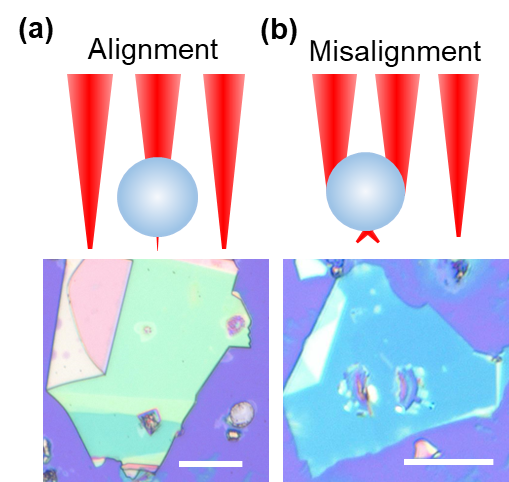


**Figure S6.** (a) Aligned and (b) misaligned microsphere-assisted femtosecond (fs) laser patterning on the hBN flakes. In (a), the microsphere is correctly positioned, leading to precise laser focusing and well-defined patterning. In (b), misalignment results in distorted or shifted laser interaction, affecting the pattern quality. Scale bar: 20 µm.

**S7. Percentage distribution of ZPL wavelengths with increasing fabrication power**


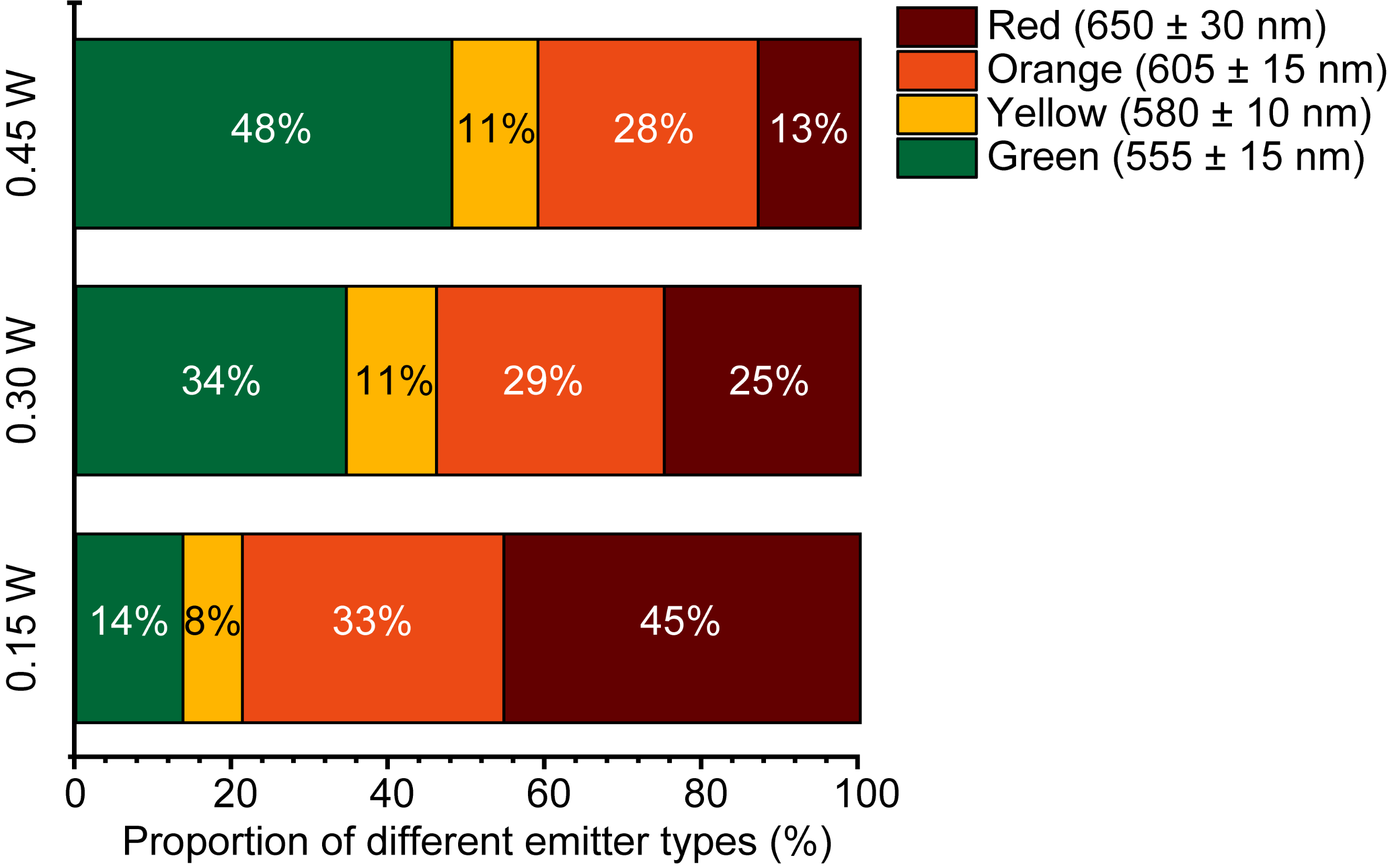


**Figure S7.** Percentage distribution of ZPL wavelengths with increasing fabrication power

**S8. Simulated electric field distribution for the emitter collection**


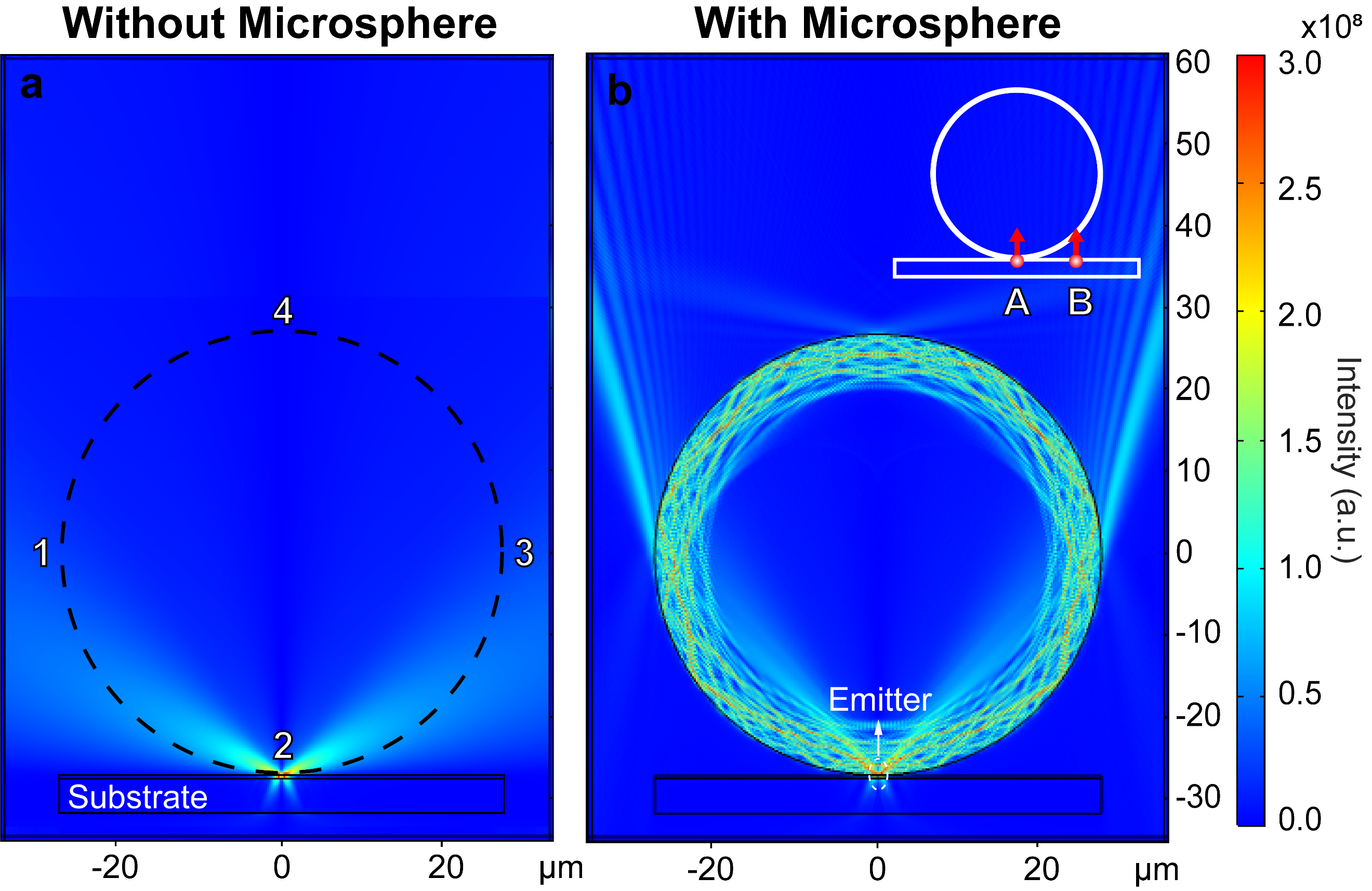


**Figure S8.** Simulated electric field distribution for the emitter collection is shown in (a) without and (b) with the MSM system. The insets display the emitter locations: at the center-bottom of the microsphere and off-center at the bottom of the microsphere, respectively.

**S9. WGM Tuning in Microspheres with Varying Refractive Indices for PL Enhancement.**


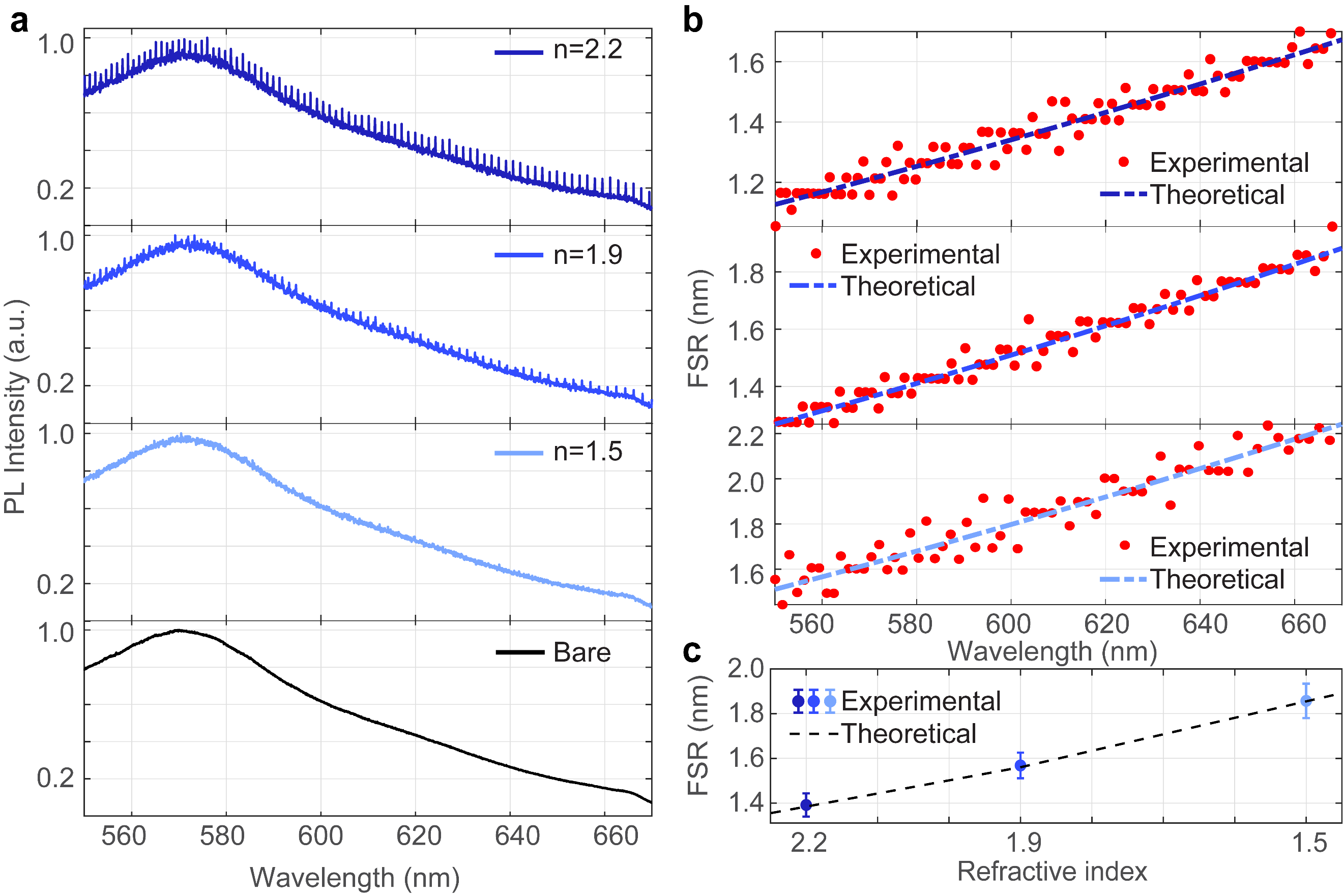


**Figure S9.** WGM Tuning in Microspheres with Varying Refractive Indices for PL Enhancement. (a) WGM-tuned PL emission spectra. (b) Experimental and theoretical of FSR with different refractive indices (c) Evolution of FSR characteristics from high to low refractive indices.

**S10. Simulated electric field distribution on the microsphere**

**Table S1.** Simulated electric field distribution at points 1,2,3 and 4 on the microsphere and without the microsphere as shown in Figure S5.


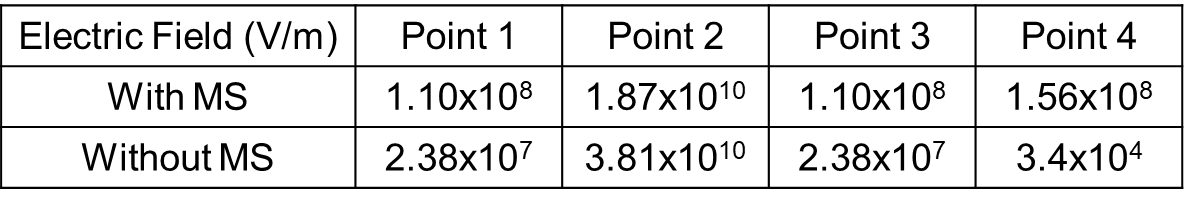


**S11. Optical images at different image planes of the microsphere**

**
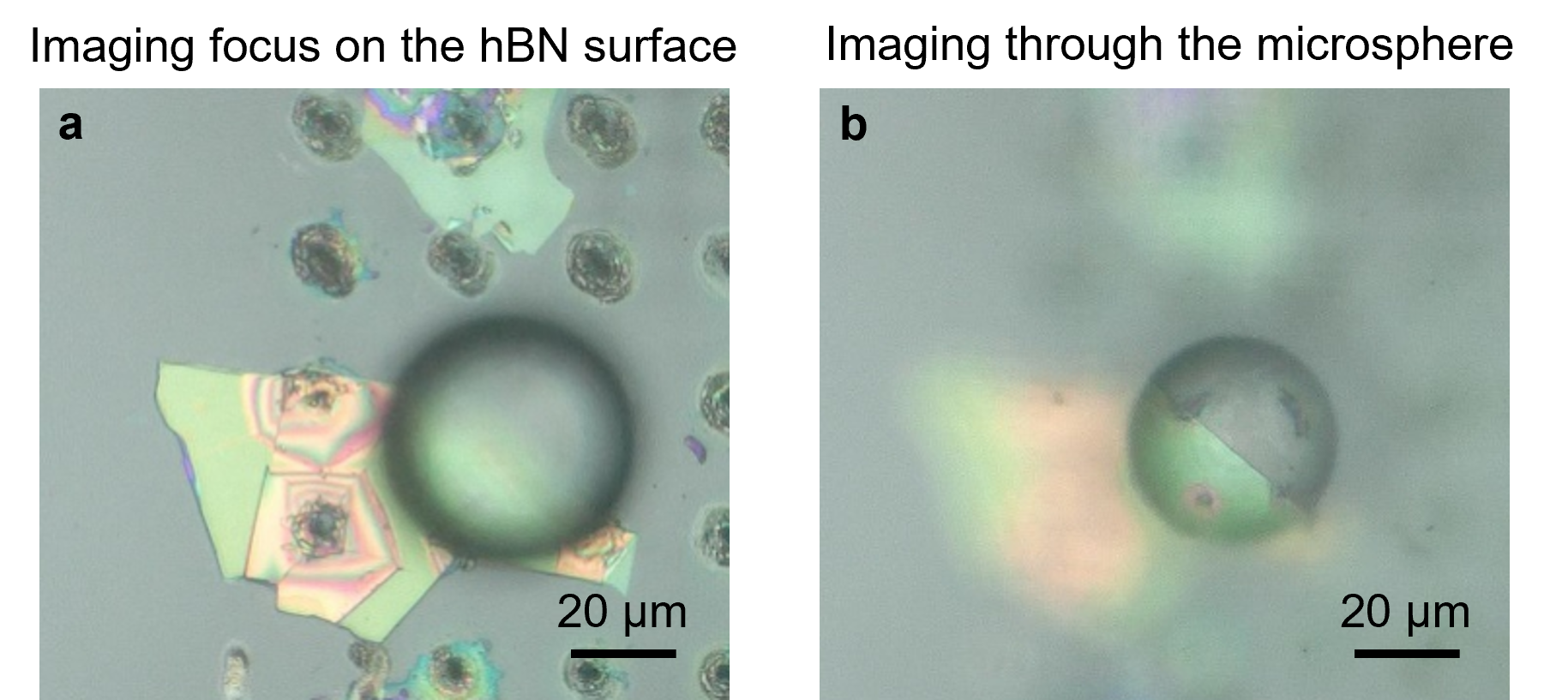
**

**Figure S10.** Optical images at different image planes of the microsphere. (a) Imaging focused on the hBN surface after fs laser fabrication. (b) Imaging of fabrication defects through the microsphere with virtual image mode.
